# Further Investigation of the Mediterranean Sponge *Axinella polypoides*: Isolation of a New Cyclonucleoside and a New Betaine 

**DOI:** 10.3390/md10112509

**Published:** 2012-11-09

**Authors:** Marialuisa Menna, Anna Aiello, Filomena D’Aniello, Ernesto Fattorusso, Concetta Imperatore, Paolo Luciano, Rocco Vitalone

**Affiliations:** 1 The NeaNat Group, Department of Chemistry of Natural Products, University of Naples “Federico II”, Via D. Montesano 49, Napoli 80131, Italy; Email: aiello@unina.it (A.A.); filomena.daniello@unina.it (F.D.); fattoru@unina.it (E.F.); cimperat@unina.it (C.I.); rocco.vitalone@unina.it (R.V.); 2 C.S.I.A.S. (Interdepartmental Center of Instrumental Analysis), University of Naples “Federico II”, Via D. Montesano 49, Napoli 80131, Italy; Email: pluciano@unina.it

**Keywords:** natural products, sponges, *Axinellidae*, pyrrole-imidazole alkaloids, taxon-specificity

## Abstract

An exhaustive exploration into the metabolic content of the Mediterranean sponge *Axinella*-*polypoides* resulted in the isolation of the new betaine **5** and the new cyclonucleoside **8**. The structures of the new metabolites were elucidated by spectroscopic methods assisted by computational methods. The analysis also provided evidence that the sponge does not elaborate pyrrole-imidazole alkaloids (PIAs) but, interestingly, it was shown to contain two already known cyclodipeptides, compounds **9** (verpacamide A) and **10**.

## 1. Introduction

**Figure 1:**
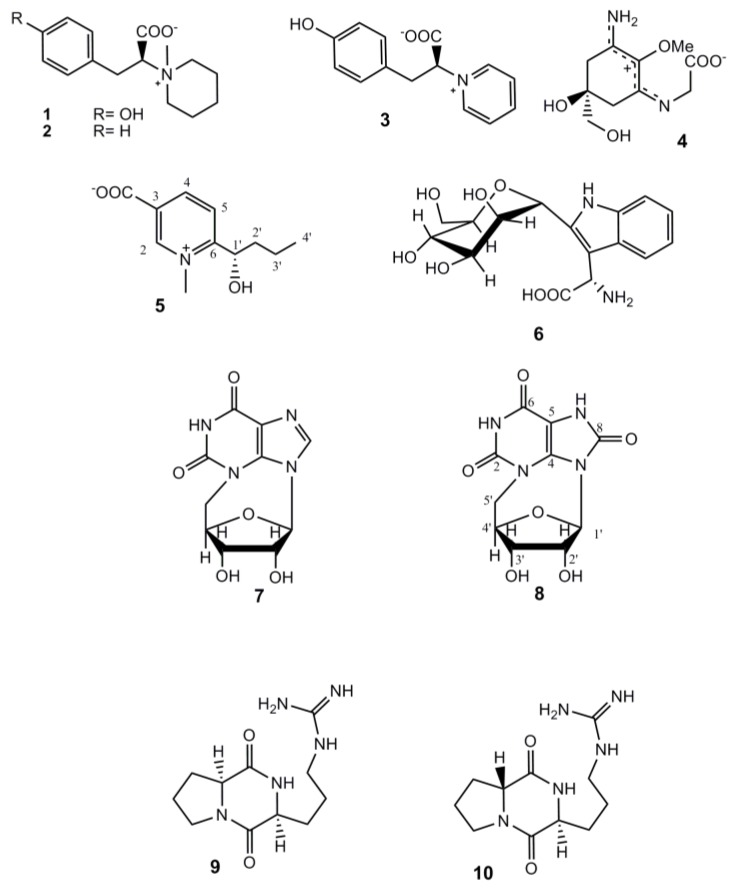
Secondary metabolites isolated from *A. polypoides*.

Diverse natural products found within sponges mediate many of their ecological interactions, including defense against predators and fouling organisms. Marine sponges of genus *Axinella* are an interesting target for chemo-ecological investigations. They are a well-known source of pyrrole-imidazole alkaloids (PIAs), which have been found only in the marine environment to date. Historically, this family of alkaloids has attracted the attention of natural product chemists because of their structural complexity and pharmacological activity; their role in chemically mediated interactions of Caribbean sponges has also been proven [1, 2]. In addition, the systematic recurrence of PIAs in *Axinellidae* sponges, as well as in *Agelasidae*, allowed for speculation as to their taxon-specificity and consideration of these secondary metabolites as chemical markers for phylogenetically related sponges [3]. However, the setting up of a chemical “fingerprint” of a sponge, collected from different ecosystems, requires exhaustive chemical studies. An illustrative example is the sponge *Axinella polypoides* (Schmidt 1862), widely distributed in the rock reefs of the Mediterranean Sea. Early studies are reported in literature on the chemistry of this sponge, mainly concerning its steroid and lectin content [4, 5, 6, 7, 8]; actually, recent re-examinations of samples of *A. polypoides*, coming from different Mediterranean areas, demonstrated that the sponge has an efficient biosynthetic potential, revealing a great variety and abundance of secondary metabolites, some of them being new molecules (Figure 1). In detail, two new modified amino acids, axiphenylalaninium (**1**) and axityrosinium (**2**), were found in specimens of the sponge collected off Marseille city [9] along with the known metabolites C^2^-α-d-mannopyranolsyl-l-tryptophan (**6**) [9, 10, 11, 12], palythine (**4**) [9, 13, 14], *N*^3^,5′-cycloxanthosine (**7**) [9, 11, 15], and taurine [9, 16]; no data was reported on the presence of PIAs in the sponge. Successively, during our ongoing search for new bioactive alkaloids from *Axinellidae* sponges, we have isolated compounds **1** and **2** in specimens of *A. polypoides* collected along Corsican coasts, together with a new betaine, polyaxibetaine (**3**) [17].

In the present communication we describe the results obtained from a further and more exhaustive exploration into the metabolic content of a larger sample of *A. polypoides*, which resulted in the isolation of a new pyridinium derivative, compound **5**, and the new cyclonucleoside **8.** All the previously reported secondary metabolites from the sponge were re-isolated, except for palythine (**4**). We did not find any member of the PIAs family in the sponge extract but, interestingly, it was shown to contain the already known cyclodipeptides **9 **(verpacamide A) and **10**. The cyclo(l-Arg-d-Pro) dipeptide **10** was first isolated from a marine bacterium, *Pseudomonas *sp. IZ208 [18]; the relevant cyclo(l-Arg-l-Pro) stereoisomer, verpacamide A (**9**), was first reported as a natural metabolite of the sponge *Axinella vaceleti* along with the analogues verpacamides B–D and these metabolites were considered as possible precursors of PIAs [19].

## 2. Results and Discussion

Specimens of *A. polypoides* were collected in the bay of Calvi (Corsica, France), they were immediately frozen after collection, and kept at −20 °C until extraction. For the extraction, fresh thawed tissues of the sponge were homogenized and exhaustively extracted at room temperature with MeOH and CHCl_3_ successively. The extracts were combined and concentrated; the resulting aqueous suspension was then partitioned between butanol and H_2_O. Both organic and aqueous layers were fractionated by MPLC over a reversed-phase C-18 column and by DCCC, respectively. All the fractions thus obtained were subjected to a combined NMR/ESIMS-based monitoring for the rapid identification of PIAs, also exploiting, as reference compounds, the copious chemical library of these alkaloids available in our laboratories. None of the already described PIAs was detected; fractions of interest were separated by repeated HPLC, resulting in the isolation of pure compounds **1**–**3 ** and **5**–**10. **Taurine, as well as compounds **1**–**3**, **6**, **7**, **9**, and **10** were readily identified by comparison of their spectroscopic data with those reported in literature [9, 10, 11, 12, 13, 14, 15, 16, 17], while the structures of the new compounds **5** and **8** were established as follows.

HRESI mass spectrum (positive ions) of **5** revealed two pseudomolecular ion peaks at *m/z* 210.1130 and 232.0950, corresponding to [M + H]^+^ (calculated value: 210.1125), and [M + Na]^+^ (calculated value: 232.0944), respectively. The molecular formula C_11_H_15_NO_3_ was thus established for **5**, indicating five unsaturation degrees. ^1^H-NMR spectrum of **5 **(CD_3_OD), interpreted on the basis of 2D experiments (HSQC, COSY) contained a set of aromatic signals, each integrating for one proton; the chemical shift and coupling constants values of these signals [δ 9.10 (bs, δ_C_ = 148.7, H-2); 8.84 (bd, *J* = 8.2 Hz, δ_C_ = 146.3, H-4); 8.19 (d, *J* = 8.2 Hz, δ_C_ = 126.6, H-5)] were strongly indicative of a 1,2,5-trisubstituted pyridinium ring. Further proton resonances were a deshielded methine at δ 5.13 (dd, *J* = 9.0, 3.3 Hz, δ_C_ = 69.3, H-1′), two AB methilene systems at δ 1.72/1.81 (δ_C_ = 38.9, 2H-2′) and 1.54/1.63 (δ_C_ = 19.9, 2H-3′), as well as a methyl resonating as a triplet at δ 1.00 (*J* = 7.5 Hz, δ_C_ = 14.0, 3H-4′), which were arranged in a single spin system on the basis of COSY connectivities. The signal at δ 5.13 was correlated in the HSQC spectrum with a signal at δ 69.3 attributable to an oxygen bearing carbon, thus evidencing a 1-hydroxybutyl unit. 

An N-methyl signal δ 4.38 (δ_C_ = 46.1) was also present in the proton spectrum of **5**; it was correlated in the ROESY spectrum (CD_3_OD) with the aromatic proton singlet at δ 9.10 (H-2) and with the oxymethine proton at δ 5.13 (H-1′). This latter information, according to the coupling constants pattern of the aromatic signals, provided convincing evidence for an N-methyl-2,5-disubstituted pyridinium ring and indicated that the 1-hydroxybutyl unit must be linked at one of the N-flanking carbons. Diagnostic C–H long range couplings, evidenced by HMBC map (CD_3_OD, see Table 1), substantiated the proposed structural features and allowed to identify the third substituent, obviously liked at C-3. 

**Table 1:** NMR data (CD_3_OD) of compound **5**.

Position	δ_H_ (mult., *J *in Hz)	δ_C_	HMBC
1-NMe	4.38 (s)	46.1	2, 6
2	9.10 (bs)	148.7	1-NMe, 4, 6, COO^-^
3	-	137.8	-
4	8.84 (bd, *8.2*)	146.3	2, 6, COO^−^Na^+^
5	8.19 (d, *8.2*)	126.6	3, 6, 1*′*
6	-	163.0	-
1*′*	5.13 (dd, *3.3*, *9.0*)	69.3	6, 2*′*
2*′*	1.72 (m)	38.9	6, 1*′*, 3*′*, 4*′*
	1.81(m)		
3*′*	1.54 (m)	19.9	1*′*, 2*′*, 4*′*
	1.63 (m)		
4*′*	1.00 (t, *7.5*)	14.0	2*′*, 3*′*
–COO^-^	-	167.0	-

Particularly, the N-methyl signal at δ 4.38 was correlated to the carbon at δ 148.7 (C-2) and a quaternary carbon at δ 163.0 (C-6) which, in turn, was coupled to the signal at δ 5.13 (H-1′) and 8.19 (H-5); the latter signal was correlated with the oxymethine carbon at δ 69.3 (C-1′) and the quaternary carbons at δ 163.0 (C-6) and 137.8 (C-3). Moreover, both aromatic signals at δ 9.10 (H-2) and 8.84 (H-4) were coupled to a low-field carbon resonance at δ 167.0, attributable to a carbonyl; this datum, according to the mass data, allowed to locate a carboxylate function at C-3, thus accounting for the last unsaturation degree indicated by the molecular formula. Therefore, the planar structure of compound **5** was unambiguously defined as 6-(1-hydroxybutyl)-1-methylpyridinium-3-carboxylate.

**Figure 2:**
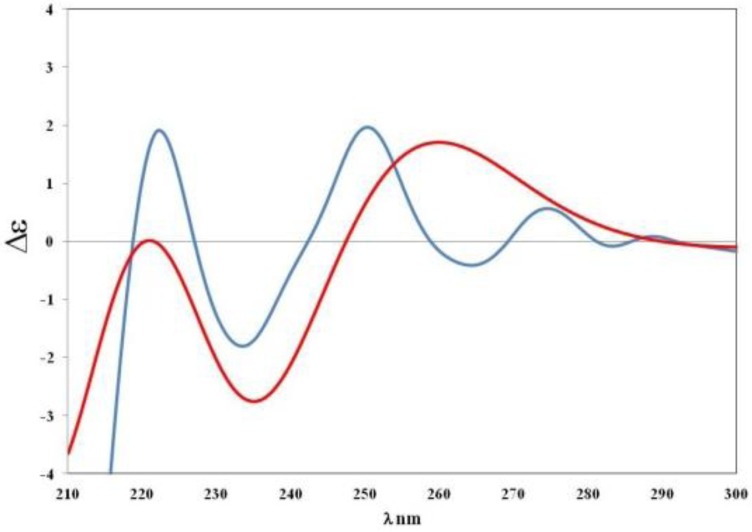
Theoretical CD curve (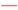) of *S*-**5** model *vs. *experimental curve (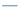) of compound **5**.

Absolute configuration (AC) of compound **5 **was established with the help of electronic circular dichroism (ECD) spectroscopy assisted by quantum mechanical calculations. The application of *ab initio* time-dependent density functional theory (TDDFT) to the calculation of ECD spectra has greatly enhanced the reliability with which they can be predicted and, thus, this methodology is being increasingly utilized in determining ACs of natural products [17, 20, 21, 22]. The experimental ECD spectrum of **5** was virtually compared to that predicted by TDDFT calculations for one of the two enantiomers. In detail, an initial conformational analysis of the *S* stereoisomer was performed, using the Simulated Annealing procedure (INSIGHT II Software Package). The resulting conformers were ranked on the basis of their conformational energy values and grouped into families. Twelve minima for *S*-**5** were obtained and all conformers were optimized with the software package Gaussian 03 [23] by using DFT at the RB3LYP/6-31G(d) level (the conformational families of *S*-**5**, as well as their resulting relative (∆E) and free (∆G) energies, are provided as Supporting Information). For all conformers of *S-***5**, the excitation energies, as well as the oscillator and rotatory strengths of the electronic excitation were calculated, using the TDDFT methodology at the RB3LYP/6-31G(d,p) level; their ECD spectra were then simulated by the overlapping Gaussian function. To obtain the final ECD spectrum of each compound, the simulated spectra of the lowest energy conformations were averaged, by following the Boltzmann statistic, and were UV corrected. The theoretical curve was then compared to the experimental spectrum of compound **5**, recorded in MeOH. As shown in Figure 2, the agreement between the simulated and experimental ECD spectra of **5 **was very satisfactory and, thus, the absolute configuration of **1 **was established as *S.*

A pseudomolecular peak was present in the HRESI mass spectrum (positive ions) of **8 **at *m/z* 283.0673 [M + H]^+^ (calculated value: 283.0679), thus supporting the molecular formula C_10_H_10_N_4_O_6_. ^1^H-and ^13^C-NMR spectral data of **8 **strongly resembled those of the known cyclonucleoside **7 **(see Table 2). The proton spectrum of **8** (*d*_6_-DMSO) contained indeed resonances relevant to a sugar moiety which were nearly identical to those of **7**, with regard to the number and shape of the signals (see Table 2); the striking difference was confined to the chemical shift value of the anomeric proton (H-1′, δ 5.67 in **8 ***versus *6.15 in **7**). As for the heterocyclic moiety, the ^1^H-NMR spectrum of **8** lacked the signal due to the single aromatic proton of xanthosine present in that of **7 **at δ 7.79 but a further exchangeable proton at δ 11.2 (NH-7) was present. On the other hand, ^13^C-NMR spectrum (*d*_6_-DMSO), interpreted also on the basis of the HSQC experiment, contained a further deshielded quaternary carbon resonance at δ 149.6 instead of the C-8 methine carbon resonating at δ 134.5 in the ^13^C-NMR spectrum of **7. **All these findings indicated the 8-oxo-*N*^3^,5-cycloxanthosine structure for compound **8**, according to MS data and HMBC correlations (see Table 2). 

**Table 2:** NMR data (*d_6_*-DMSO) of compounds **7** and **8**.

Position	7		8		
	δ_C_	δ_H_ (*J* in Hz)	δ_C_	δ_H_ (*J* in Hz)	HMBC ^a^
1	-	-	-	-	-
2	151.0	-	150.7	-	-
3	-	-	-	-	-
4	140.7	-	136.8	-	-
5	117.8	-	98.8	-	-
6	157.4	-	153.4	-	-
7	-	-	-	-	-
8	134.5	7.79 (s)	149.6	-	-
9	-	-	-	-	-
1*′*	92.5	6.15 (s)	91.1	5.67 (s)	4, 8, 2*′*, 3*′*, 4*′*
2*′*	75.8	3.87 (dd, 5.2, 5.0)	75.0	3.90 (t, 5.3)	1*′*, 4*′*
3*′*	70.1	4.20 (m)	70.7	4.23 (m)	5*′*
4*′*	83.7	4.55 (m)	83.6	4.46 (m)	-
5*′*a	51.3	4.56 (m)	52.3	4.51 (dd, 14.4, 2.4)	2, 4, 3*′*, 4*′*
5*′*b	51.3	3.71 (dd, 15.2, 3.2)	52.3	3.72 (dd,14.4, 2.8)	2, 4, 3*′*, 4*′*
OH-2*′*	-	5.62 (d, 5.0)	-	5.60 (d, 5.3)	-
OH-3*′*	-	5.62 (bs)	-	5.35 (d, 7.1)	-
NH-1	-	11.2	-	11.3	5, 6
NH-7	-	-	-	11.2	4, 5, 8

^a^ HMBC correlations are from proton(s) stated to the indicated carbon.

## 3. Experimental Section

### 3.1. General Experimental Procedures

HRESIMS (positive mode) were performed on a Thermo LTQ Orbitrap XL mass spectrometer. The spectra were recorded by infusion into the ESI source using MeOH as the solvent. Optical rotations were measured with a Perkin-Elmer 192 polarimeter at 589 nm using a 10 cm microcell. ECD spectra were recorded on an J-710 spectropolarimeter (Jasco, Tokyo, Japan) equipped with a J-710 for Windows software (Jasco). ^1^H (700 MHz and 500 MHz) and ^13^C (175 MHz and 125 MHz) NMR spectra were recorded on a Varian INOVA spectrometer; chemical shifts were referenced to the residual solvent signal (CD_3_OD: δ_H_ = 3.31, δ_C_ = 49.0; *d*_6_-DMSO: δ_H_ = 2.50, δ_C_ = 39.0). Homonuclear ^1^H connectivities were determined by COSY experiments. Through-space ^1^H connectivities were evidenced using a ROESY experiments with a mixing time of 500 ms. Two and three bond ^1^H-^13^C connectivities were determined by gradient 2D HMBC experiments optimized for a ^2,3^*J *of 8 Hz.

### 3.2. Collection, Extraction and Isolation

Specimens of *A. polypoides *were collected in the Bay of Calvi (Corsica, France), frozen immediately and kept frozen until extraction. A reference specimen was deposited at the Dipartimento di Chimica delle Sostanze Naturali, University of Naples “Federico II”. Fresh thawed animals (105.2 g dry weight after extraction) were homogenized and extracted twice with MeOH and, then, twice with CHCl_3_ (4 × 500 mL). Extracts were combined and concentrated; the resulting aqueous residue was then partitioned between H_2_O and *n*-BuOH. Separation of the organic phase (13.0 g) was achieved by reversed-phase silica gel (RP18) MPLC, using a gradient elution (H_2_O→MeOH→CHCl_3_). Ten fractions (A–L) were obtained, each of them was subjected to a rapid ^1^H-NMR/ESIMS-based analysis. Fraction B, eluted with H_2_O/MeOH 9:1 v/v, (3.4 g) was re-chromatographed under medium pressure on a RP18 column eluting with a linear gradient of MeOH (from 2% to 100%) in H_2_O, thus affording twelve fractions (1–12). Fraction 8 (219 mg), eluted with H_2_O/MeOH 92:8 v/v, was separated by HPLC on a Synergy Polar-RP 4 μm column (250 × 4.60 mm) eluting with H_2_O/MeOH (98:2, v/v) and 0.1% TFA, to give compounds **7** (22.3 mg) and **8** (4.0 mg). Fraction 10 (55.8 mg), eluted with H_2_O/MeOH (85:15, v/v) was separated by HPLC in the same conditions as above, to give **9** (7.8 mg) and **10** (4.8 mg). Fractions C and D, both eluted with H_2_O/MeOH 7:3 v/v, were combined (524.5 mg) separated by HPLC on a Synergy Polar-RP 4 μm column (250 × 4.60 mm) eluting with H_2_O/MeOH (98:2, v/v), to give axityrosinium (**1**, 52.0 mg), axiphenylalaninium (**2**, 19.2 mg), polyaxibetaine (**3**, 6.0 mg) and compound **6** (9.1 mg).

The hydrophilic extract (42.7 g) was subjected to Droplet Counter Current Chromatography (DCCC), using a mixture of BuOH-Acetone-H_2_O (3:1:5) as the solvent, in the ascending mode. The flow rate of the mobile phase was adjusted to 25 mL/h; a total of 150 fractions of 8 mL each were collected and, as for the organic extract, analyzed by ^1^H-NMR/ESIMS for the rapid detection of PIAs. Fractions 40–50 were combined, the solvent was evaporated, and the residue (300 mg) was chromatographed by HPLC on a Synergy Polar-RP 4 μm column (250 × 4.60 mm) eluting with H_2_O/MeOH (98:2, v/v) and 0.1% TFA, thus affording pure **5** (16.4 mg).

Compound **5**: [α]_D_ +7.59 (MeOH, *c* = 0.005; HRESI-MS (positive ion mode): *m/z* = 210.1130 [M + H]^+^, 232.0950 [M + Na]^+^; ^1^H and ^13^C NMR data (CD_3_OD) are reported in Table 1. 

Compound **8**: [α]_D_ +1.70 (MeOH, *c* = 0.003); HRESI-MS (positive ion mode): *m/z* = 283.0673 [M + H]^+^, 265.0567 [M − H_2_O + H]^+^; ^1^H and ^13^C NMR data (*d*_6_-DMSO) are reported in Table 2.

### 3.3. Computational Details

A preliminary conformational search for one of the two enantiomers of **5 **was performed by Simulated Annealing in the INSIGHT II package. The MeOH solution phases were mimicked through the value of the corresponding dielectric constant. Using the steepest descent followed by quasi-Newton-Raphson method (VA09A) the conformational energy was minimized. Restrained simulations were carried out for 500 ps using the CVFF force field as implemented in Discover software (Accelrys, San Diego, USA). The simulation started at 1000 K, and then the temperature was decreased stepwise to 300 K. The final step was again the energy minimization, performed in order to refine the structures obtained, using the steepest descent and the quasi-Newton-Raphson (VA09A) algorithms successively. Both dynamic and mechanic calculations were carried out by using 1 (kcal/mol)/Å 2 flat well distance restraints. One hundred structures were generated. To simulate the solvent chosen for NMR analysis, a distance-dependent dielectric constant set to the value of MeOH (ε 32.63) was used during the calculations. All optimizations were performed with the software package Gaussian 03, by using the DFT functional RB3LYP and the basis set 6-31G(d). The B3LYP/6-31G(d) harmonic vibrational frequencies were further calculated to confirm their stability. Rotatory strength values for the electronic transitions from the ground state to the singly excited states for all conformers of *S*-**5 **were obtained by TDDFT calculations RB3LYP/6-31G(d,p) with Gaussian 03. The rotatory strength values were summed after a Boltzmann statistical weighting and ∆ε values were calculated by forming sums of Gaussian functions centered at the wavelengths of the respective electronic transitions and multiplied by the corresponding rotatory strengths. The ECD spectra that was obtained was UV-corrected and compared with the experimental one.

## 4. Conclusions

While highlighting the uncommon chemodiversity of *A. polypoides*, these results pointed out that the sponge does not elaborate PIAs. This finding is in agreement with the results of a recent investigation on the chemical defense of *A. polypoides* and the co-occurring species *A. verrrucosa*, against microbial fouling and in feeding deterrence of a potential predator [24], activities which are believed to be mediated by PIAs [1, 2]. This study indicated in *A. polypoides* neither chemical defense against microbial fouling nor chemically mediated feeding deterrence activity. In contrast, the sympatric sponge *A. verrucosa* has a chemical defense which, as in many other species of the genera *Axinella *and *Agelas*, is mediated by its bromopyrrole compounds [25, 26, 27], such as hymenidin, which exhibits multiple defensive roles [24]. Recent studies have reported on the phylogenetic status of the genus Axinella [28], which is difficult to define on the basis of its morphological character and includes a heterogeneous assemblage of species. A new phylogenetic hypothesis of Axinellidae and Axinella, based on two independent molecular markers (18S and 28S rRNA), has been proposed. In this taxonomic reconstruction, *A. polypoides* and *A. vaceleti* belong to a distinct clade (Axinellidae^P^) than *Axinella verrucosa*; this latter sponge is now included in a new clade, named *Cymbaxinella*^P^, which could be interpreted also as a new genus. The new clade *Cymbaxinella*^P^ constitutes a well supported clade sister-group to *Agelas*^P^, according to the chemotaxonomic hypothesis proposed by Braekman *et al.* [29] on the basis of the presence of pyrroles. The clades *Agelas*^P^ and *Cymbaxinella*^P^ constitute a new clade: *Agelasida*^P^. Our results, thus, provide a chemotaxonomic support for the above mentioned molecular analysis, demonstrating that *A. polypoides* does not contain pyrroles, whereas it shares with *A. vaceleti* the presence of diketopiperazine derivatives. 

## Supplementary Files

Supplementary File 1: PDF-Document (PDF, 1160 KB)
