# Supplementary material for: Further Investigation of the Mediterranean Sponge Axinella polypoides: Isolation of a New Cyclonucleoside and a New Betaine
Source: Mar Drugs. 2012 Nov 9;10(11):2509–18. doi: 10.3390/md10112509 (PMC3509532; doi:10.3390/md10112509)
Supplement: Supplementary File 1: — PDF-Document (PDF, 1160 KB) [file marinedrugs-10-02509-s001.pdf]

Supplementary Information

Figure S1. ^{1}H-NMR spectrum of compound **5**. 2
Figure S2. COSY spectrum of compound **5**. 3
Figure S3. HSQC spectrum of compound **5**. 4
Figure S4. HMBC spectrum of compound **5**. 5
Figure S5. ^{13}C-NMR spectrum of compound **5**. 6
Figure S6. HRESI mass spectrum of compound **5**. 7
Figure S7. ^{1}H-NMR spectrum of compound **8**. 8
Figure S8. COSY spectrum of compound **8**. 9
Figure S9. HSQC spectrum of compound **8**. 10
Figure S10. HMBC spectrum of compound **8**. 11
Figure S11. HRESI mass spectrum of compound **8**. 12
Figure S12. ^{13}C-NMR spectrum of compound **8**. 13
Figure S13. Minimum Energy Conformations for *S*-**5**. 14
Figure S14. Calculated ECD of *S*-**5** at the B3LYP/6-31G **. 15
Table S1. Conformational Analysis of *S*-5 conformers in methanol. 16

Figure S1. ^{1}H-NMR spectrum of compound **5**.

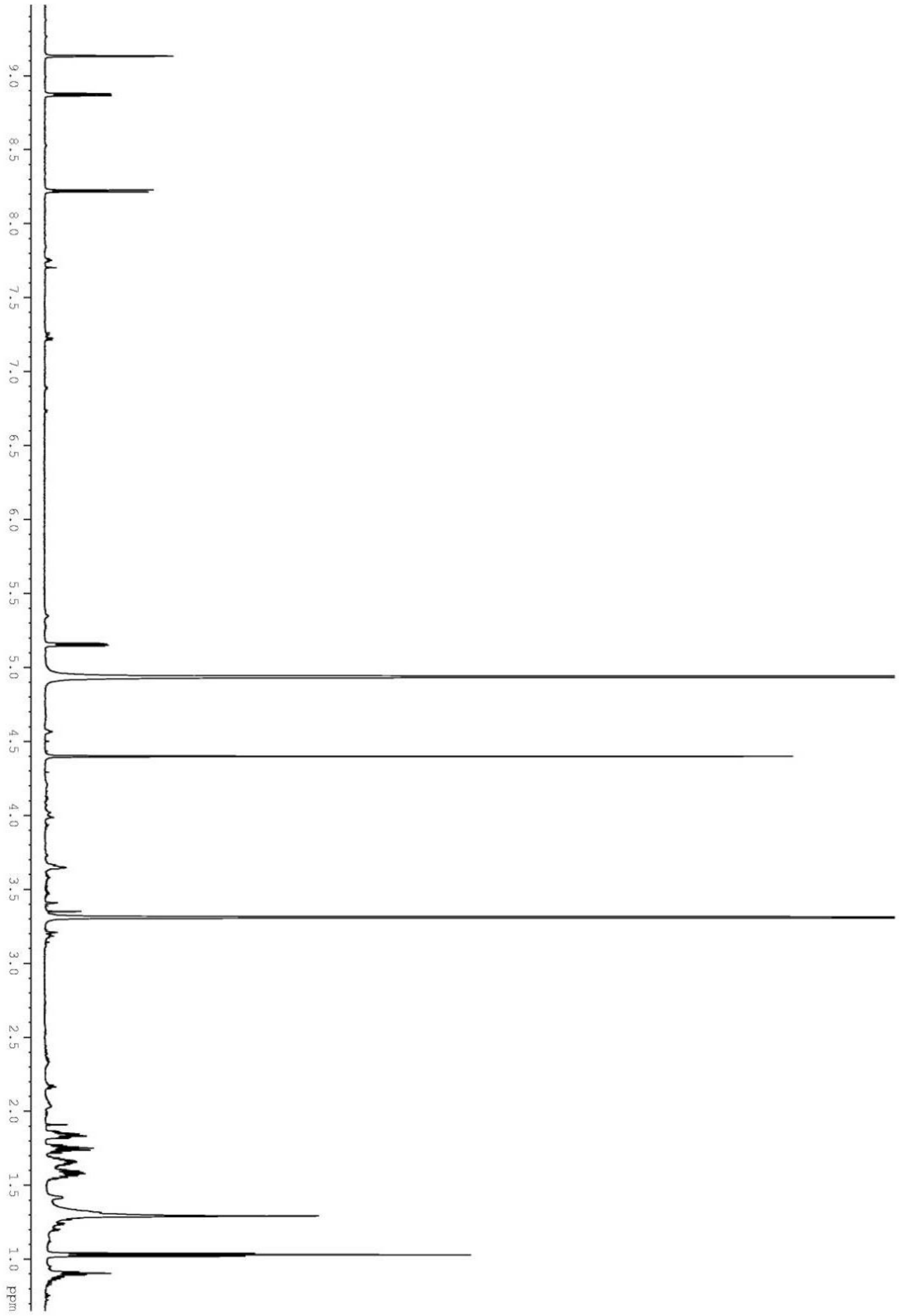

Figure S2. COSY spectrum of compound **5**.

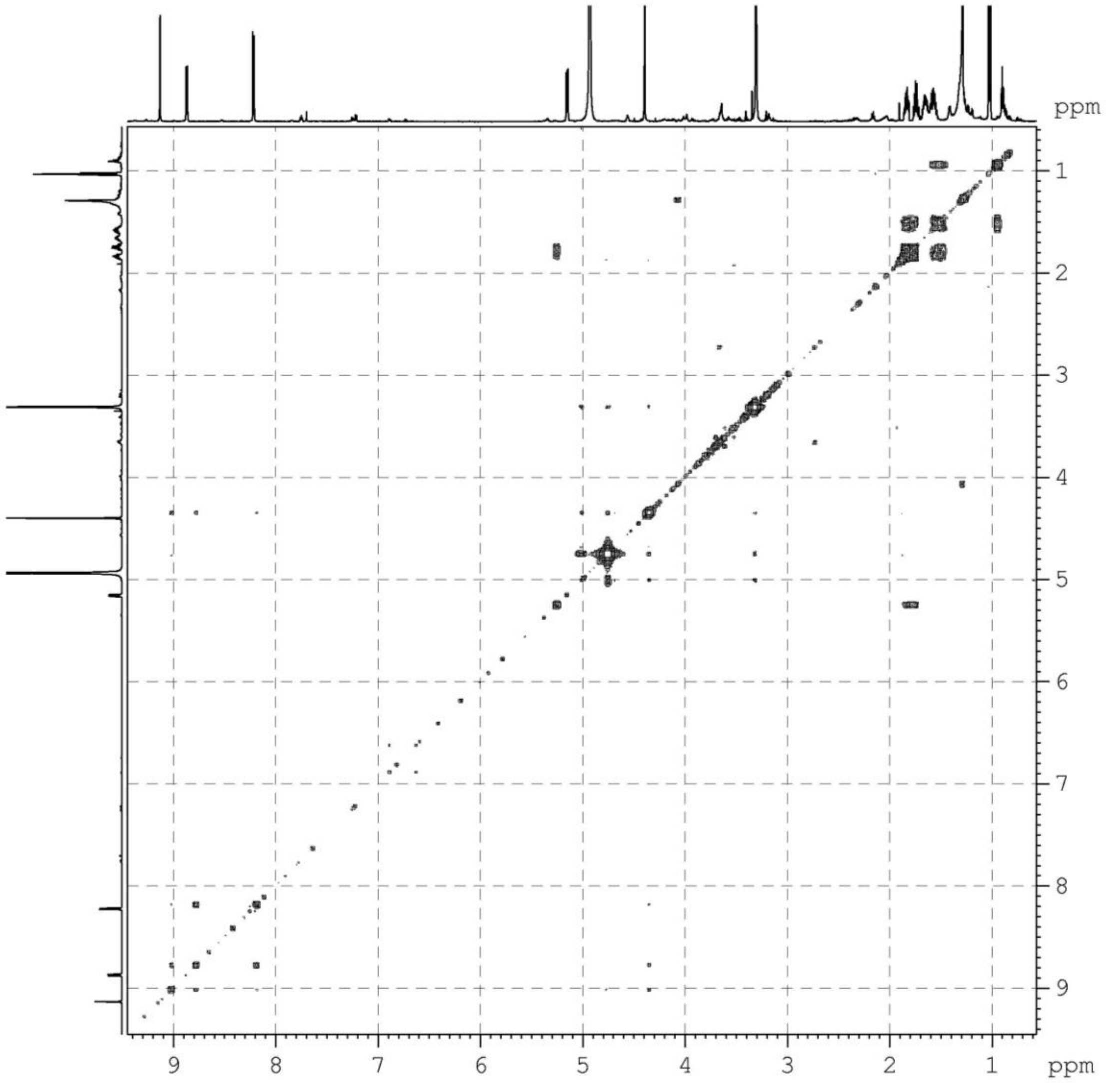

Figure S3. HSQC spectrum of compound **5**.

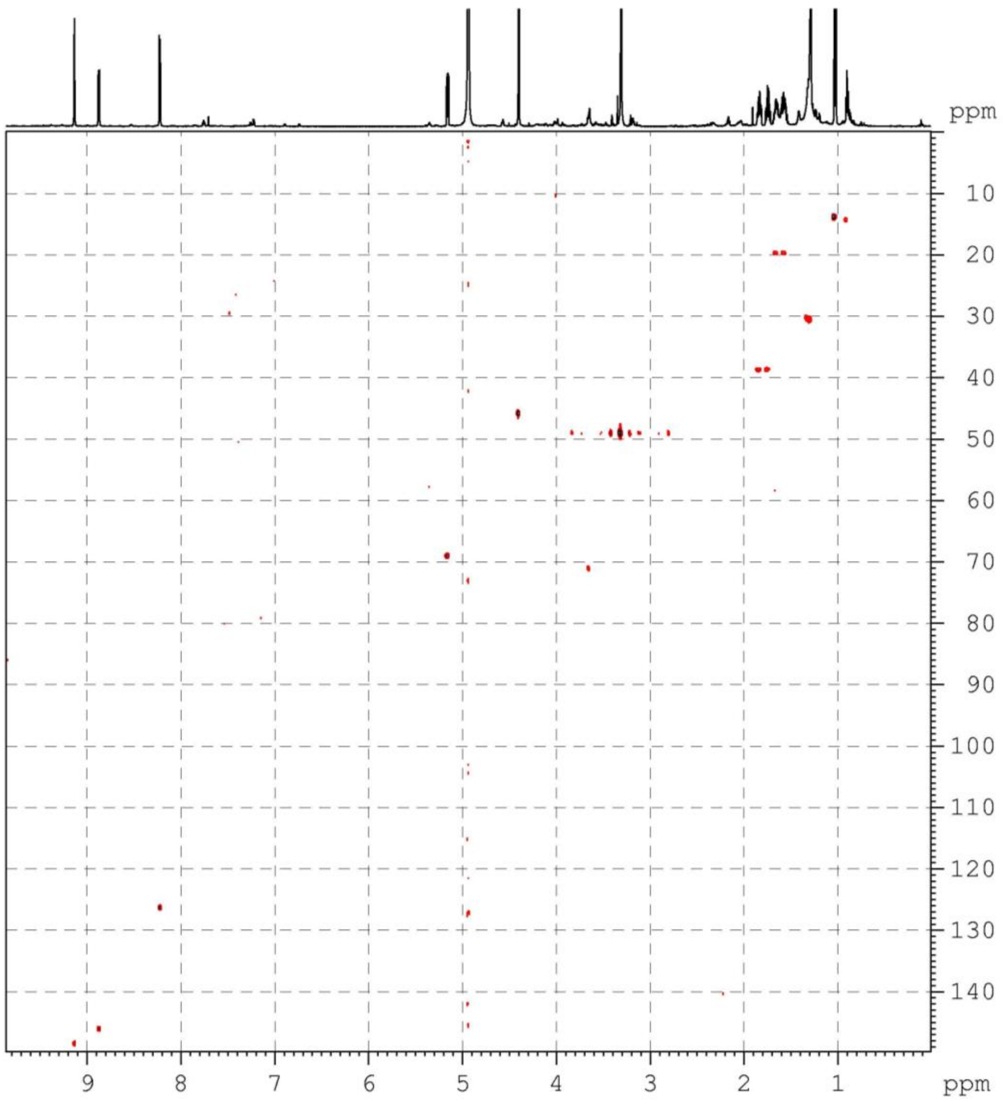

Figure S4. HMBC spectrum of compound **5**.

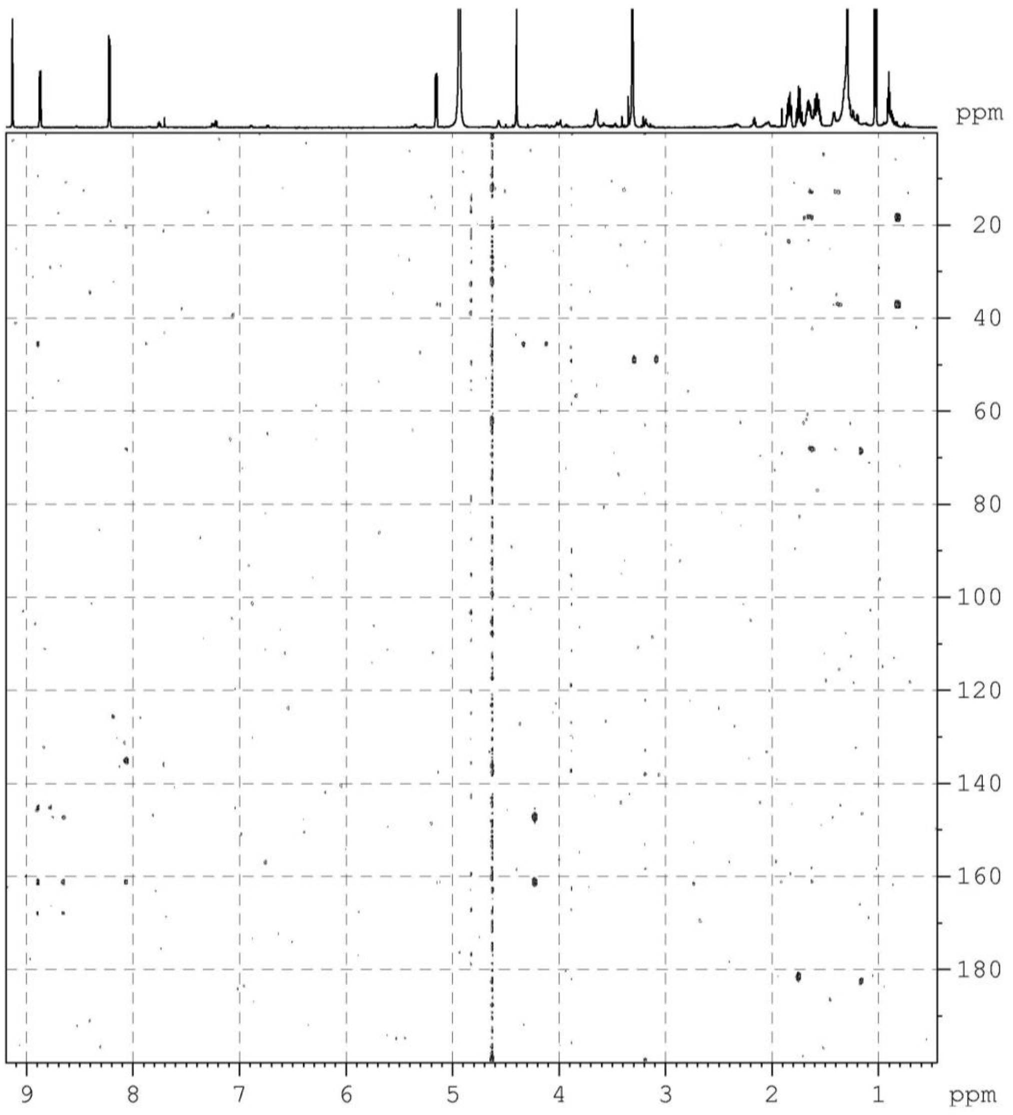

Figure 5. ^{13}C-NMR spectrum of compound **5**.

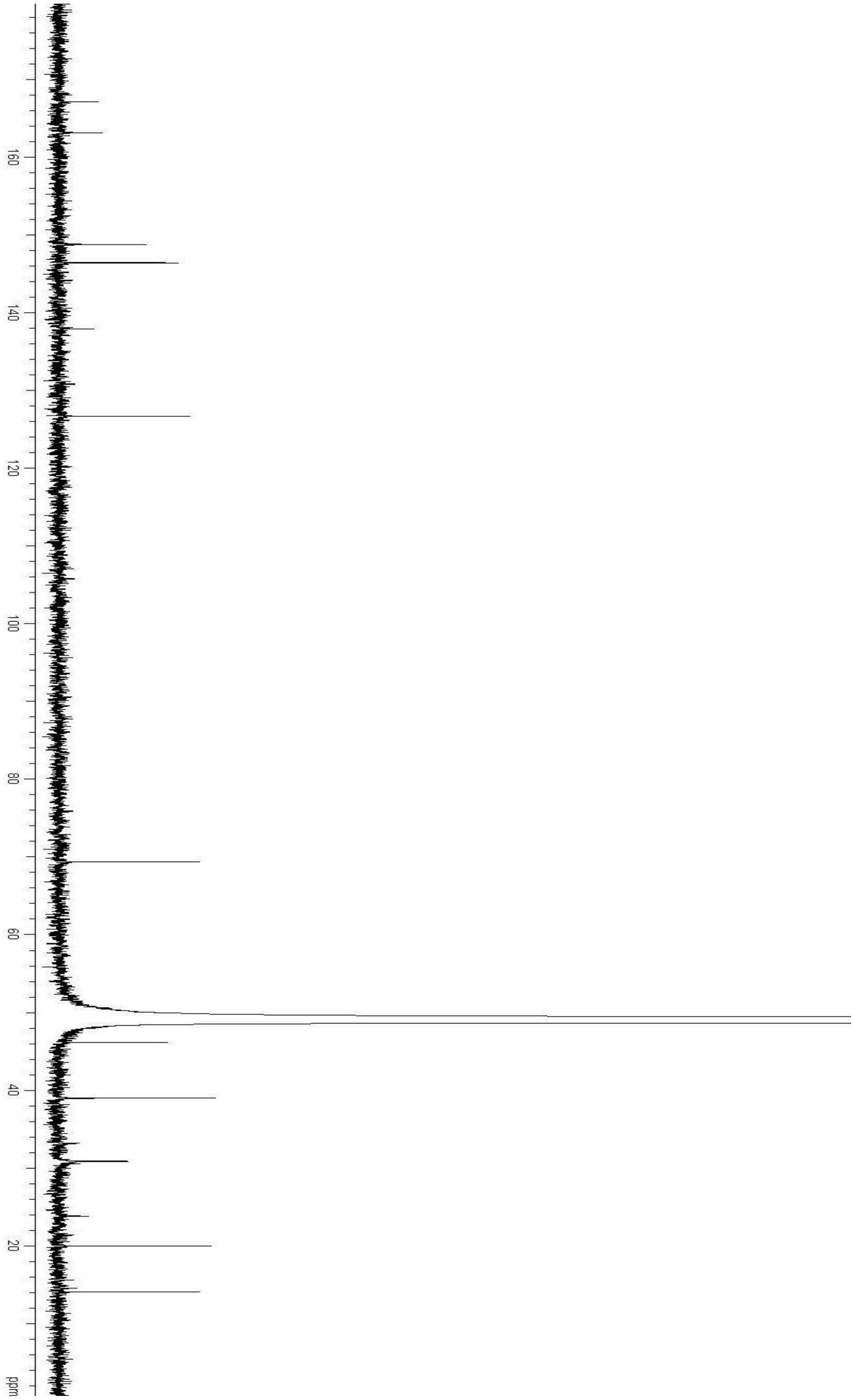

Figure S6. HRESI mass spectrum of compound **5**.

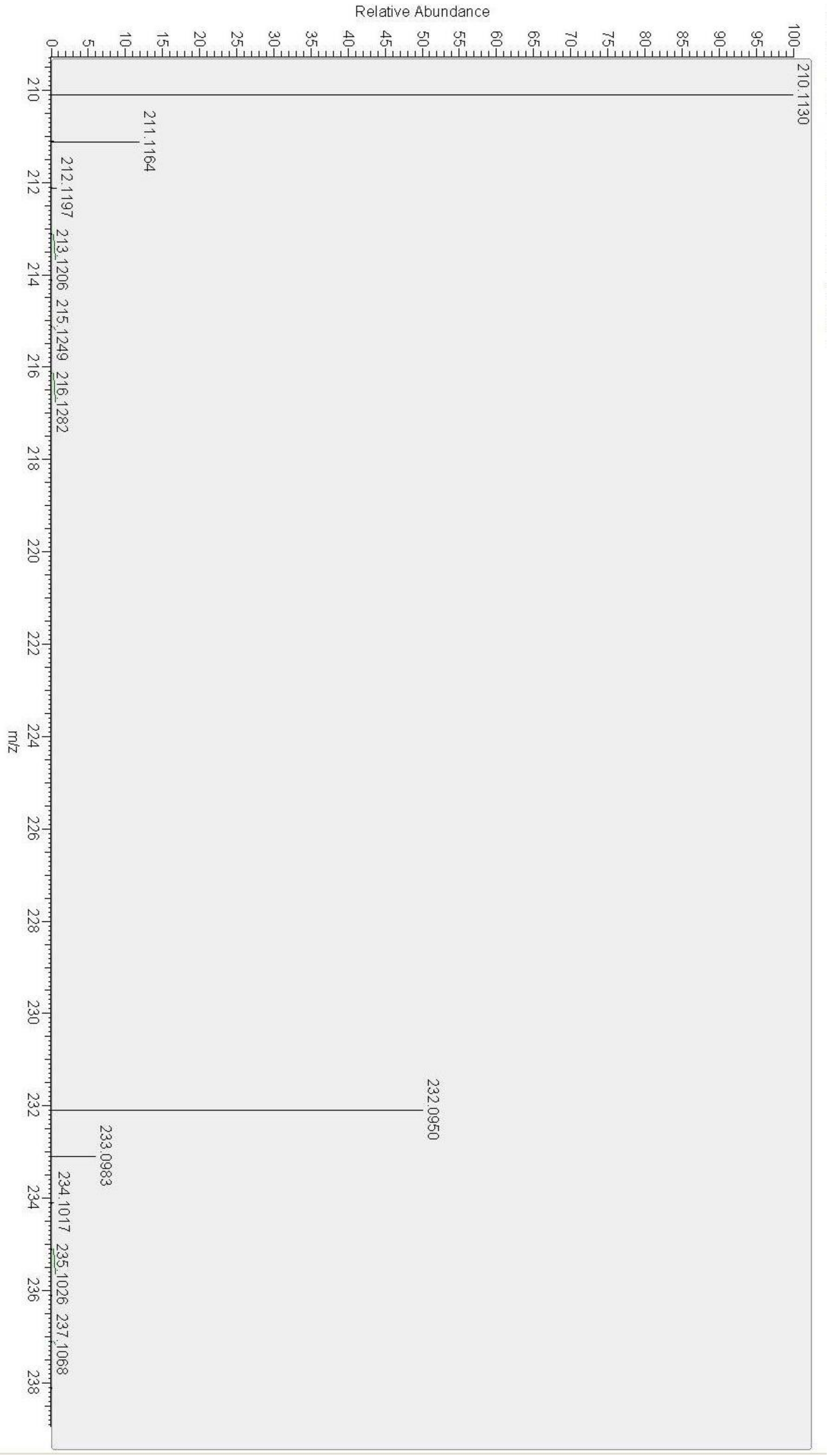

Figure S7. ^{1}H-NMR spectrum of compound **8**.

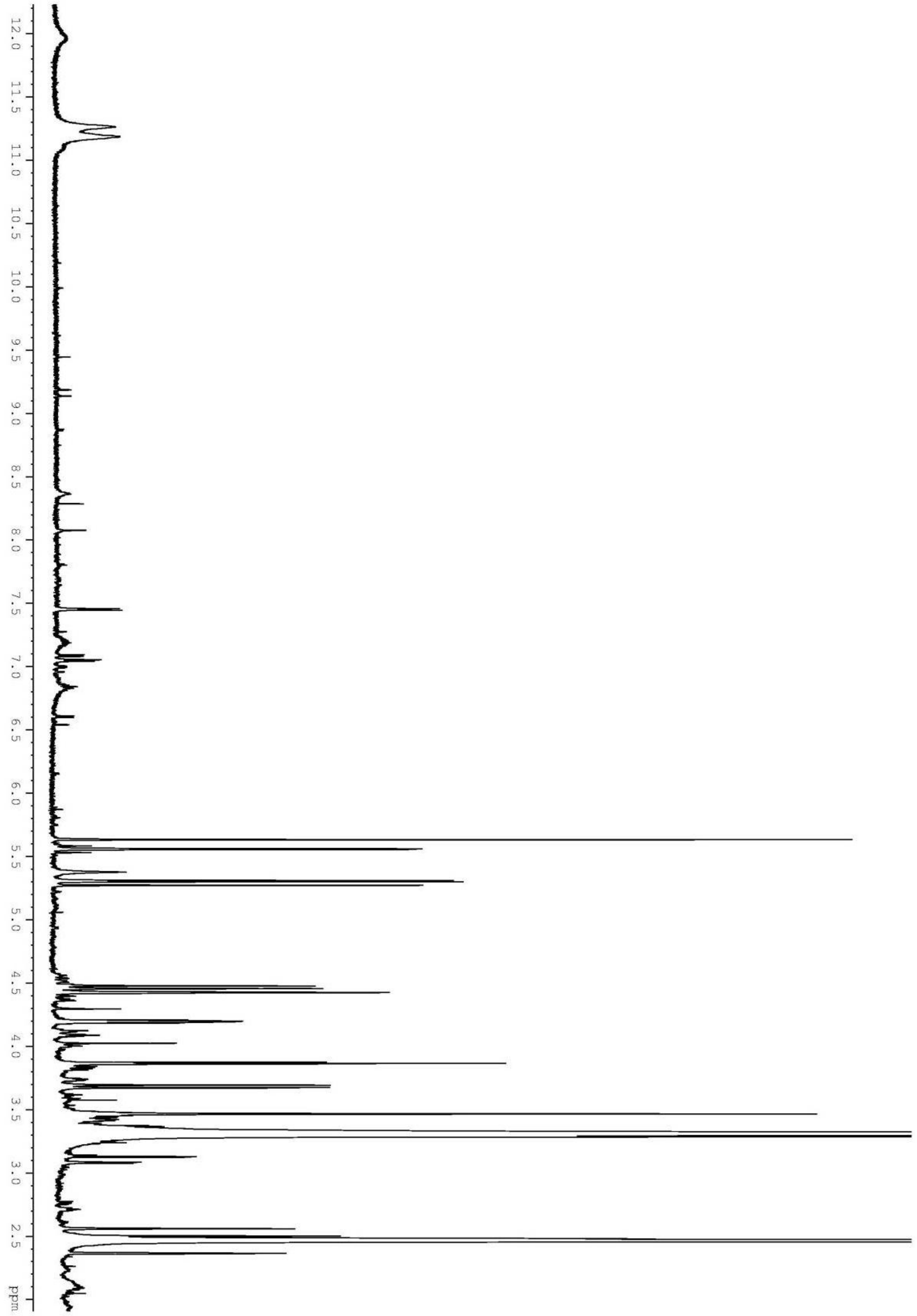

Figure S8. COSY spectrum of compound **8**.

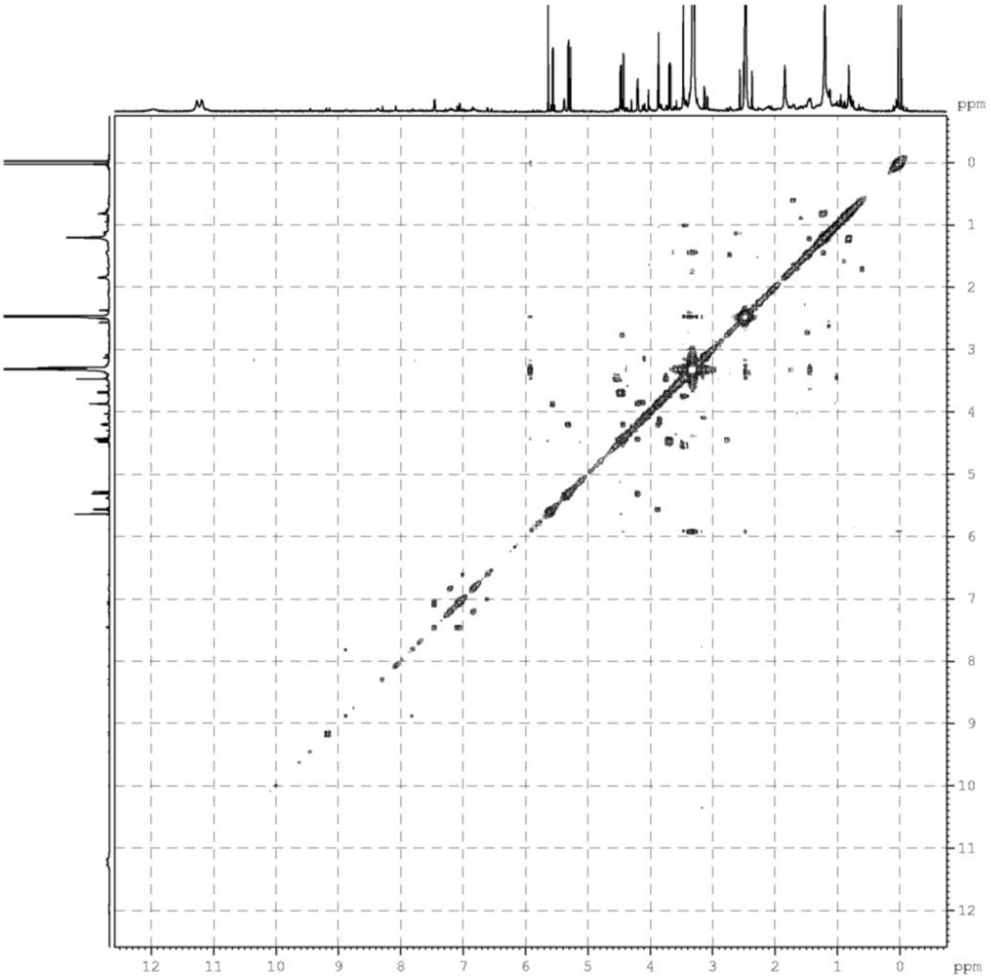

Figure S9. HSQC spectrum of compound **8**.

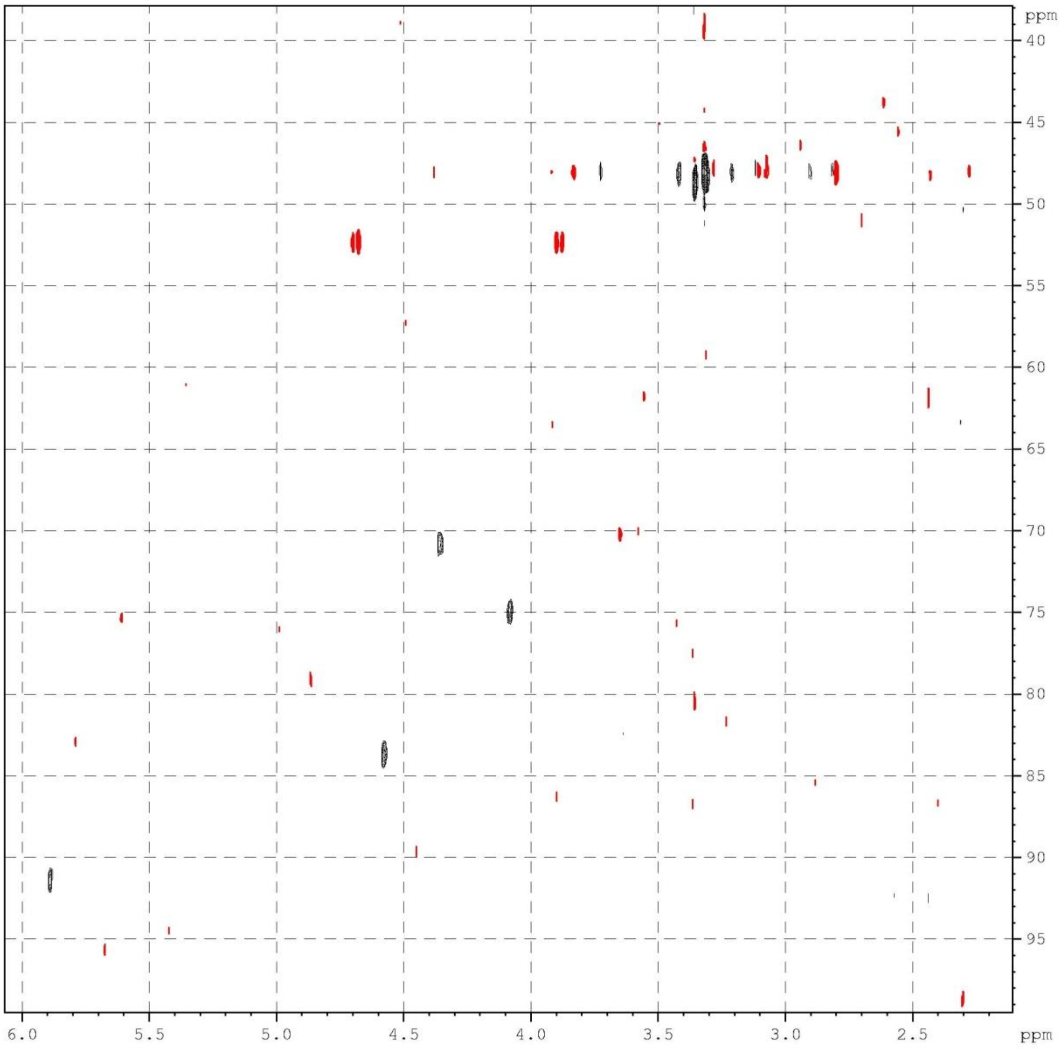

Figure S10. HMBC spectrum of compound **8**.

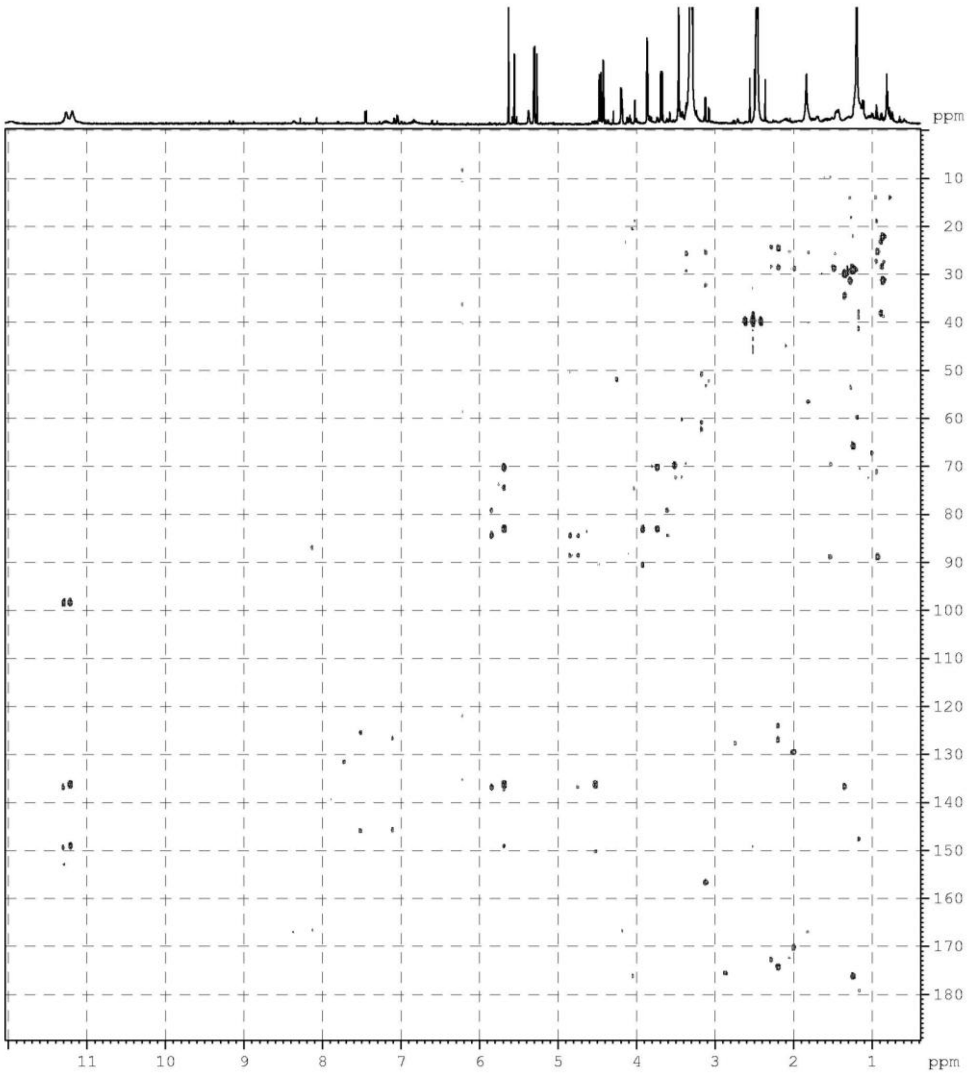

Figure S11. HRESI mass spectrum of compound **8**.

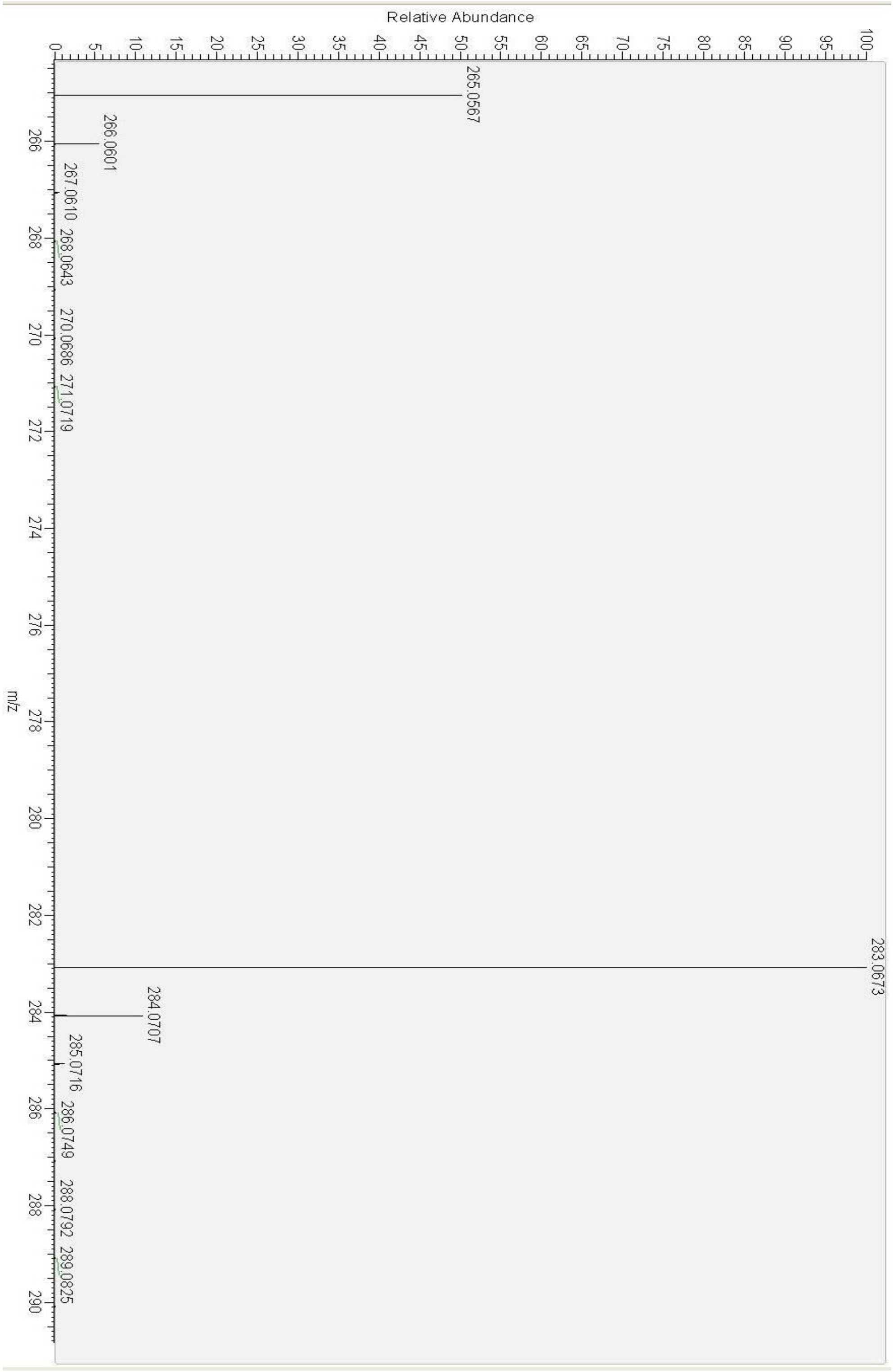

Figure S12. ^{13}C-NMR spectrum of compound **5**.

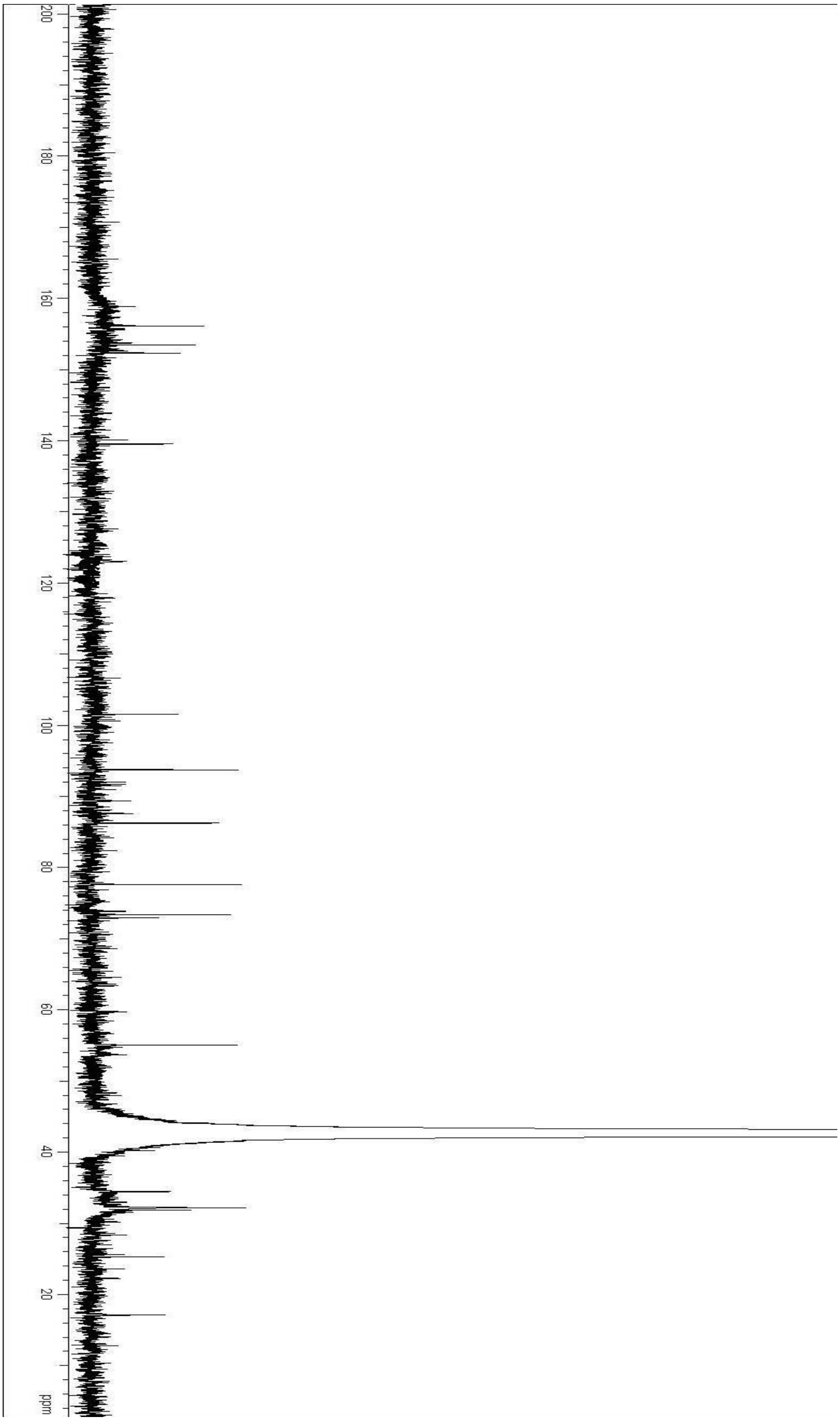

Figure S13. Minimum Energy Conformations for *S*-**5**.

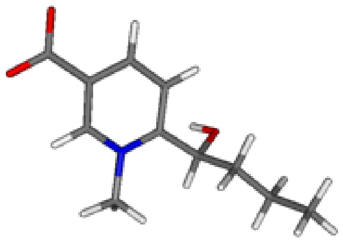
S-**5**a

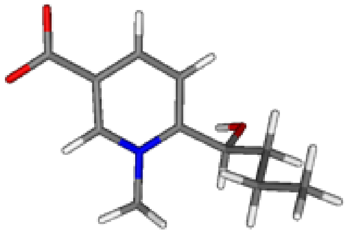
S-**5**b

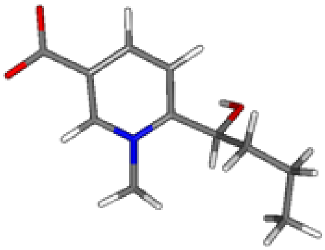
S-**5**c

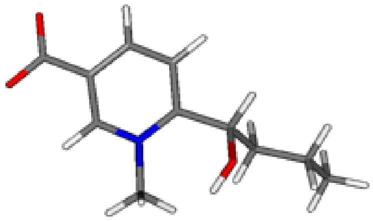
S-**5**d

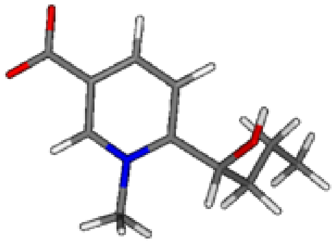
S-**5**e

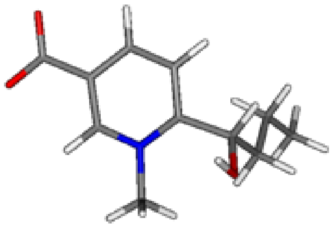
S-**5**f

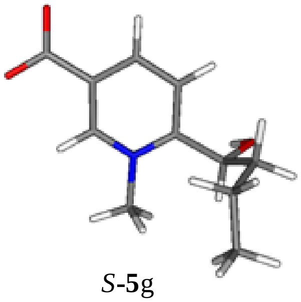
S-**5**g

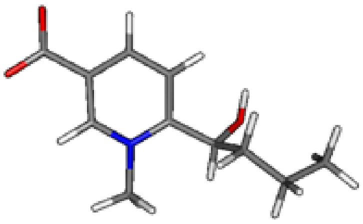
S-**5**h

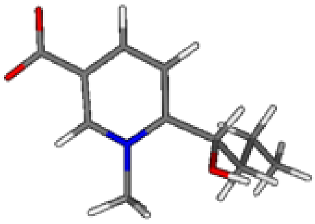
S-**5**i

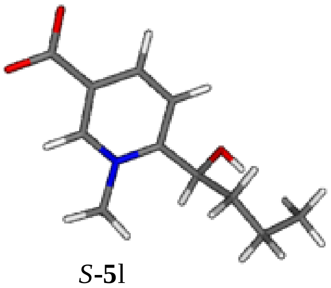
S-**5**l

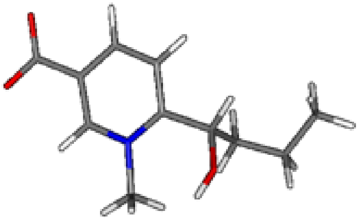
S-**5**m

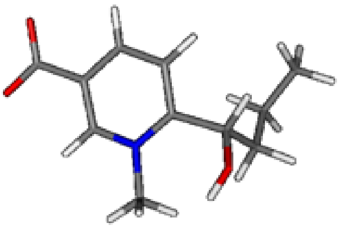
S-**5**n

Figure S14. Calculated ECD of *S*-**5** at the B3LYP/6-31G **.

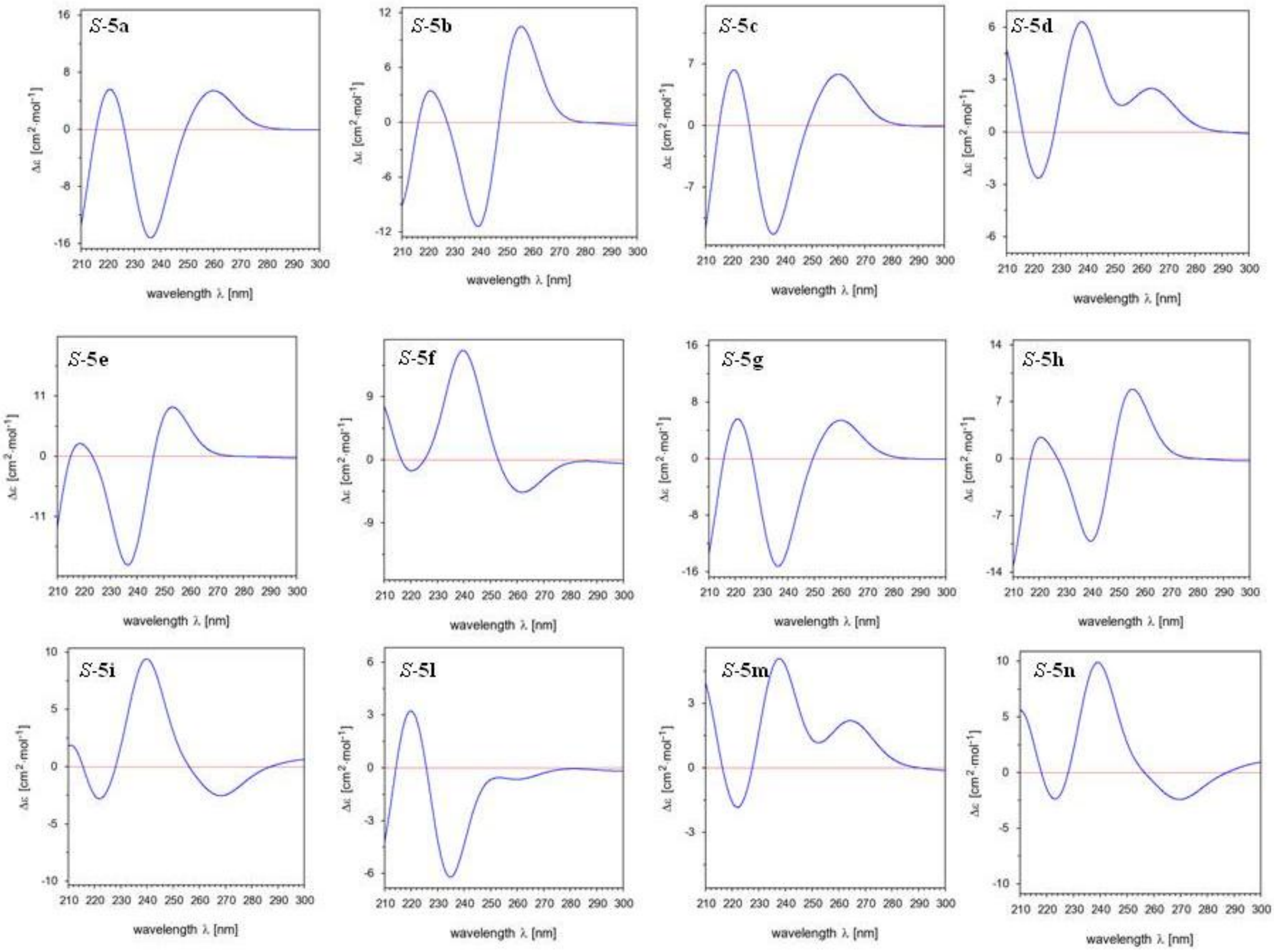

Table S1. Conformational Analysis of *S*-5 conformers in methanol.

	**E** [a]	**G** [b]	**P%** [c]
S-**5a**	0.00	0.00	45.75
S-**5b**	0.25	0.52	18.91
S-**5c**	0.79	0.69	14.29
S-**5d**	0.91	1.01	8.32
S-**5e**	1.05	1.25	5.55
S-**5f**	1.24	1.57	3.23
S-**5g**	1.26	1.62	2.97
S-**5h**	1.47	3.08	0.25
S-**5i**	1.47	3.08	0.25
S-**5l**	1.81	3.25	0.19
S-**5m**	1.81	3.25	0.19
S-**5n**	1.94	3.69	0.09

[a] Relative energy (kcal/mol). [b] Relative Gibbs free energy (kcal/mol). [c] Conformational distribution calculated at the at the B3LYP/6-31G(d) level in methanol.
